# Greater Early Disambiguating Information for Less-Probable Words: The Lexicon Is Shaped by Incremental Processing

**DOI:** 10.1162/opmi_a_00030

**Published:** 2020-03-01

**Authors:** Adam King, Andrew Wedel

**Affiliations:** Department of Linguistics, University of Arizona; Department of Linguistics, University of Arizona

**Keywords:** language efficiency, Zipf’s law of abbreviation, incremental processing, language evolution, information theory

## Abstract

There has been much work over the last century on optimization of the lexicon for efficient communication, with a particular focus on the form of words as an evolving balance between production ease and communicative accuracy. Zipf’s law of abbreviation, the cross-linguistic trend for less-probable words to be longer, represents some of the strongest evidence the lexicon is shaped by a pressure for communicative efficiency. However, the various sounds that make up words do not all contribute the same amount of disambiguating information to a listener. Rather, the information a sound contributes depends in part on what specific lexical competitors exist in the lexicon. In addition, because the speech stream is perceived incrementally, early sounds in a word contribute on average more information than later sounds. Using a dataset of diverse languages, we demonstrate that, above and beyond containing more sounds, less-probable words contain sounds that convey more disambiguating information overall. We show further that this pattern tends to be strongest at word-beginnings, where sounds can contribute the most information.

## BACKGROUND

Human languages are characterized by hierarchically organized, nested structure: utterances are composed of structured sequences of words, and words in turn are composed of structured sequences of sounds. Many of the ways these structures are organized in language have been argued to result in more efficient transmission of information than would occur otherwise (e.g., Fedzechkina, Jaeger, & Newport, 2012; Ferrer i Cancho, 2017; Futrell, Mahowald, & Gibson, 2015; Genzel & Charniak, 2002; Gibson et al., 2019; Gildea & Jaeger, 2015; Hale, 2003, 2006; Hawkins, 2010; Jaeger, 2010; Jaeger & Tily, 2011; Levy, 2008), suggesting that the details of language structures evolve under pressure to optimize communicative efficiency. The lexicon—roughly, the set of words in a language—is one of these possible loci of optimization, and can be conceptualized as a code that maps meaningful lexical units (referred to here as *words*) to word-forms, for example, a sequence of sounds, or *segments*. The relationship between words and their word-forms is not fixed a priori, but can evolve over the course of language change, such as when the original compound *electronic mail* shortened to *email* with increasing use. Because the lexicon is a constantly evolving system, and because many lexical properties of interest—such as word length—can be straightforwardly measured, the lexicon has been a focus for much prior research on the role of biases toward efficient communication in shaping language patterns. Many of these studies conclude that patterns in the lexicon support the hypothesis that communicative efficiency is a driving pressure in the evolution of word to form mappings (Ferrer i Cancho & Solé, 2003; Kanwal, Smith, Culbertson, & Kirby, 2017; Piantadosi, Tily, & Gibson, 2009, 2012; Zipf, 1949).

One of the most cross-linguistically robust observations in this domain is Zipf’s law of abbreviation: more-probable words tend to be shorter, while words that are less probable tend to be longer (Bentz & Ferrer i Cancho, 2016; Piantadosi, Tily, & Gibson, 2011; Zipf, 1935). In his *Principle of Least Effort*, Zipf (1949) proposed that this pattern arises as a trade-off between a pressure for accuracy on the one hand, and lower effort on the other. As it stands, there is robust evidence that the segment composition of word-forms is shaped for lower production-effort beyond the effect of short length (Dautriche, 2015; Dautriche, Mahowald, Gibson, & Piantadosi, 2017; Mahowald, Dautriche, Gibson, & Piantadosi, 2018; Meylan & Griffiths, 2017). Here we investigate whether segment composition may also be optimized to provide listeners greater disambiguating information as they identify words in the speech stream.

We can conceptually divide the information available to listeners in word identification into two sources: (a) the listener’s prior expectation that the word will occur, and (b) the information provided by the word-form itself (reviewed in Hall, Hume, Jaeger, & Wedel, 2016). If word-forms evolve under pressure to balance accuracy and effort, the amount of information from these two sources should tend to trade-off: words that are on average more probable should evolve word-forms that contain less informative material because they can do so without compromising accuracy, and conversely, words that are less probable should evolve word-forms that convey relatively more information.

All things being equal, a word-form with more segments is likely to possess more information overall. However, segments can differ in how much information they contribute to disambiguating a word-form from others: a segment in a word-form that disambiguates from many other forms in the lexicon provides more information than one that disambiguates from few. Further, earlier segments in a word-form tend to contribute more disambiguating information in word identification than later segments because listeners process word-forms incrementally, progressively updating inferences about the intended word as the segment sequence unfolds in time (e.g., Allopenna, Magnuson, & Tanenhaus, 1998; Magnuson, Dixon, Tanenhaus, & Aslin, 2007; Marslen-Wilson, 1987; see Dahan & Magnuson, 2006, and Weber & Scharenborg, 2012, for review). For example, consider the word *vacuum* /vækjum/. Perception of the word-initial [v] is highly informative as it allows a listener to begin to discount the large set of lexical items that begin with other segments. The final [m] contributes less information because the previous segments [vækju…] already indicate *vacuum* as the most likely word. Correspondingly, psycholinguistic studies show that listeners preferentially allocate attention to word-beginnings, which has been attributed to the greater information provided by early segments (Connine, Blasko, & Titone, 1993; Grosjean, 1996; Marslen-Wilson & Zwitserlood, 1989; Nooteboom, 1981; Salasoo & Pisoni, 1985).

Two related predictions for efficient lexical structure arise from the fact that different segments can convey different amounts of disambiguating information. First, words that are on average less probable should tend to not only have *longer* forms, but to have forms with relatively *higher information* segments. Second, if the lexicon is structured to capitalize on incremental word processing, this association between segmental information and word probability should be strongest early in word-forms and decay at later positions. Early segments more strongly narrow the range of lexical possibilities, and in parallel, narrow the prior contextual differences in word probability (for more on the benefit of early informativeness, see Hawkins, 2010). A useful way to think of this second prediction is that segments should become distributed throughout the lexicon such that the probability mass of competing words drops more steeply for less-probable words during processing. This can potentially be achieved with two conceptually distinct strategies, one focusing on the segmental *network structure* of the lexicon, that is, the specific sequences of segments that distinguish words, and the other on the relative word probabilities within the competing groups of words that exist.

In the first strategy, the lexicon evolves such that the segments in less-probable words act to disambiguate from a greater *number* of competing words early on. As an example, the form for the less-probable word *sphinx* begins with a nearly unique cluster, [sf], which immediately disambiguates it from most of the lexicon. In the second, the lexicon evolves such that less-probable words have segments that disambiguate from relatively high-probability competitors. Both of these strategies have the effect of reducing the probability mass of competitors faster for less-probable words.

Here, we explore these predictions, showing evidence that the lexicons of a diverse set of languages are in fact structured to be an efficient code given incremental processing, both in terms of the structure of the lexicon and the relative probabilities of competitors in that structure.

## METHODS

We investigated the relationship between segment information and word probability in phonemically transcribed corpora for 20 languages (see Table 1 in the Supplemental Materials; King & Wedel, 2020). The dataset is reasonably typologically diverse, drawn from 10 different language families from four continents, where 13 of the 20 languages are non-Indo-European. All corpora except Hausa, Kaqchikel, Malay and Tagalog were morphologically annotated, allowing us to focus the analysis on uninflected word stems. For each language, we limited our investigation to the 10,000 most frequent word-types.

We used a context-free measure of word probability (see Equation 1) which allowed us to include languages with fewer and less detailed linguistic resources.^1^

$$p(word)=\frac{count(word)}{\sum_{word^{\prime }}count(word^{\prime })}$$ (1)

Prior work on the effects of incremental word processing has measured *segment information* as the −log_2_ conditional probability of a segment given the current *cohort* (Marslen-Wilson & Welsh, 1978), that is, the set of word-forms in the lexicon that share identical segments until that point. For example, the information of [f] in *sphinx* is determined by dividing the token-count of words beginning with [sf] by the token-count of words beginning with [s], and then taking the base-2 logarithm of the resulting quantity. This token-based measure (Equation 2) has been previously shown to predict variation in information in the speech signal: lower token-based segment information correlates with shorter segment duration and less-distinct articulation (Tang & Bennett, 2018; van Son & Pols, 2003; van Son & van Santen, 2005), and lower average token-based segment information correlates with a greater probability of segment deletion in casual speech (Cohen Priva, 2015, 2017). Below, we will show that a parallel type-based measure provides similar results.

$$h(seg_{n})=-{\text{log}}_{2}\frac{count(seg_{1}...seg_{n})}{count(seg_{1}...seg_{n-1})}$$ (2)

We emphasize that this general approach for estimating the segmental information available to a listener is coarse-grained relative to how the speech stream is actually processed. For one, this measure treats segments as equivalently distinctive, abstract symbols, rather than as phonetic signals, which are differentially perceptually distinctive from one another and differentially robust to noise (Mielke, 2012; Smits, Warner, McQueen, & Cutler, 2003). We anticipate that future work will benefit from using measures that take perceptual distance into account (e.g., Gahl & Strand, 2016; Strand, 2014). Our current method also implies that there is no uncertainty in perception, assuming listeners immediately discard alternatives not compatible with each successive segment. Instead, there is evidence that lexical access is moderately tolerant of segmental mis-ordering (Toscano, Anderson, & McMurray, 2013) and that listeners can backtrack to some degree when new segmental information is incompatible with previous information (Gwilliams, Linzen, Poeppel, & Marantz, 2018; McMurray, Tanenhaus, & Aslin, 2009). Nonetheless, the method we use here should capture some portion of the information flow during lexical processing and has the advantage of being broadly applicable. We anticipate that more fine-grained, perceptually sophisticated measures will provide yet clearer outcomes.

## RESULTS

### Mean Segmental Information

Less-probable word-forms tend to have more overall segmental information just by virtue of having more segments (Piantadosi et al., 2011; Zipf, 1935). If all segments contributed equivalent information to word identification, addition or subtraction of segments would be the only way to change the information carried by a word-form. If this were the case, the *mean* information contributed by each segment across a word-form would not correlate with word probability in words of the same length. Conversely, if we find that less-probable words have higher mean segment information when controlling for length, it suggests these words, in addition to having more segments, have more disambiguating information packed into those segments.

We begin with the token-based measure of segment information described above because it is sensitive to both categorical cohort structure and word frequencies in those cohorts (see below for parallel tests using a type-based measure). However, the token-based measure carries a built-in correlation between word probability and segment information, because the frequency of a word contributes to the calculation of information of its segments. To eliminate this source of correlation, for the results presented in this section we used a modified form of the equation in which we subtract a word’s frequency from the calculation of the information of its own segments (Equation 3).

$$h^{*}(seg_{n})=-{\text{log}}_{2}\frac{count(seg_{1}...seg_{n})-count(word)}{count(seg_{1}...seg_{n-1})-count(word)}$$ (3)

We calculated the mean segment information for each word-form including only the segments before the *uniqueness-point* (cf. Marslen-Wilson & Welsh, 1978), that is, the point at which it is the only remaining word in the cohort.^2^ We excluded post-uniqueness segments because, by this method, they contribute zero information. As a result, if segment information is averaged over the whole word, words with longer post-uniqueness-point sequences systematically show lower mean segment information values. Note, however, the relationship between word probability and mean segment information in this dataset remains significant when post-uniqueness-point segments are included (not shown), indicating that less-probable words have more incrementally informative segments on average across the entire word-form (cf. Mahowald et al., 2018; Meylan & Griffiths, 2017, who show that less-probable words are composed of phonotactically less-probable sequence types).

**Figure 1.**
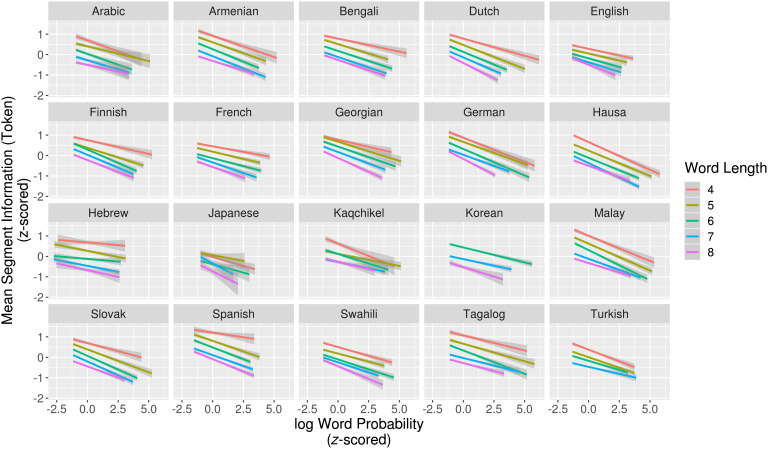
**Relationship between log word probability and mean token-based segment information for words of length 4–8.** Grayed area represents 95% confidence intervals. Less-probable words contain higher information segments.

Figure 1 shows best-fit regression lines for mean token-based segment information by word probability for word lengths 4–8 in each language. As predicted, less-probable words contain more informative segments. Across all languages, all but three (94/97) of the by-length regression models show a significant correlation between word probability and mean segment information. When word length categories are pooled and word length is included as a separate factor, the models show a significant effect of word probability for all languages (Table 2 in the Supplemental Materials; King & Wedel, 2020).^3^

Using a token-based measure for segment information, the *sum* of the information contributed by each segment in a word-form is equal to context-free word information, −log_2_*p*(*word*).^4^ As a consequence, the negative correlation that we find between *mean* segment information and word probability can only arise if the information in segments of less-probable words are concentrated in fewer segments. This has the effect of more rapidly reducing the probability mass of alternatives with each successive segment.

There are two conceptually distinct ways this more rapid reduction in probability mass could be accomplished: (a) successive segments could categorically reduce the number of competitors more quickly, or (b) successive segments could preferentially eliminate higher probability cohort members. In the following sections we show evidence that lexicons are optimized in both ways.

### Segments in Less-Probable Words Reduce the Number of Competitors More Quickly

#### Cohort Size.

To ask whether the segment sequences of less-probable words tend to more rapidly disambiguate them from a greater number of competing word-types early in processing, we used a type-based variant of the measure of segment information (Equation 2). For example, the type-based information of [f] in *sphinx* is equal to the number of word-types that begin with [sf] in a corpus divided by the number that begin with [s].

**Figure 2.**
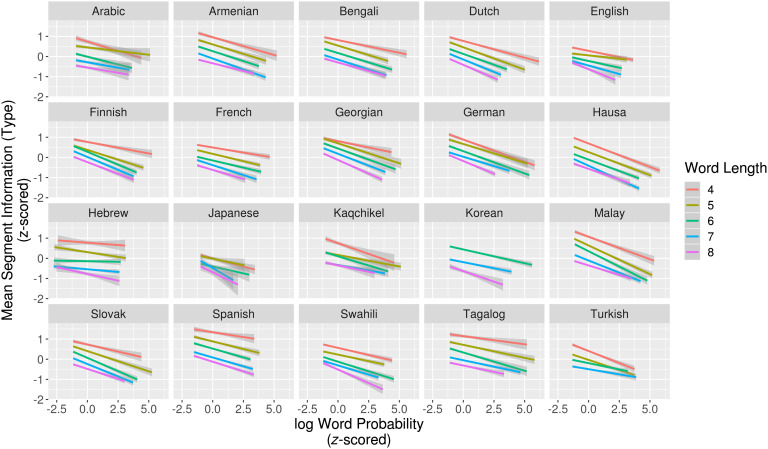
**Relationship between mean type-based segment information and log word probability for words of length 4–8.** Less-probable words more quickly reduce the cohorts of competing words.

As above, we fit linear regression models to predict word probability given mean type-based segment information for words of length 4–8 separately in all languages (Figure 2). In all but two (95/97) cases, word probability showed significant negative correlation with mean type-based segment information. Again, when all lengths were pooled together, word probability showed a significant, negative correlation with mean type-based segment information in all languages (see Table 3 in the Supplemental Materials; King & Wedel, 2020). Because the mean type-based segment information measure ignores word frequency, its significant correlation with word probability indicates that segments in less-probable words disambiguate from relatively more competitors, reducing cohort sizes more rapidly.

#### Position of Uniqueness-Point.

If less-probable words contain segments that more quickly reduce cohort sizes, then the point at which their word-forms become unique should be earlier, relative to length. As an example, the words *thwart* and *story* both have five segments, but the uniqueness-point of the less-probable *thwart* comes in its second segment (i.e., no other word in our English corpus begins with [θw]), while the uniqueness-point of *story* falls in its last segment, where it disambiguates from *stork*, *storm*, *storage*, and so on.

**Figure 3.**
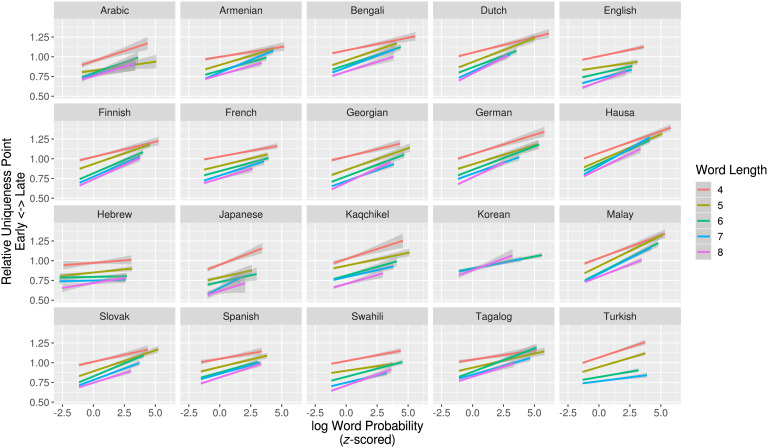
**Relationship between log word probability and relative position of uniqueness-point for words of length 4–8.** Less-probable words have relatively earlier uniqueness-points for all lengths.

Linear regression models predicting the relative position of a word’s uniqueness-point (i.e., uniqueness-point divided by number of segments) by word probability for lengths 4–8 separately showed that less-probable words do in fact have significantly earlier uniqueness-points in all but four (93/97) by-length regressions (Figure 3). As above, when lengths are pooled, we found that less-probable words have significantly earlier relative uniqueness-points in all languages (Table 4 in the Supplemental Materials; King & Wedel, 2020). When included as a factor in models to predict the nonrelative uniqueness-point position, we found an independent, positive effect of word length, suggesting that longer words have later uniqueness-points, on average (Table 5 in the Supplemental Materials; King & Wedel, 2020; cf. Strauss & Magnuson, 2008).

### Segments in Less-Probable Words Eliminate More-Probable Competitors

#### Comparison to Word Probability-Shuffled Baselines.

Here we ask whether the significant relationship between word probability and token-based segment information is in part because less-probable words tend to be grouped in cohorts with more-probable words, allowing early segments in less-probable words to eliminate a greater probability mass of competitors, independently of *how many* competitors they eliminate. To do this, we compared the real-world lexicons of each of our tested languages against probability-randomized but structurally identical variants of the lexicon, created by shuffling the context-free probability for words of the same length in each language and then recalculating segmental information.^5^ Shuffling word probabilities within length classes creates variant lexicons in which the (potentially optimized) probability relationship between words in cohorts is severed, while maintaining both the original cohort structure as well as the original relationship between word probability and length. For example, in a shuffled variant of the English lexicon, *thwart* might take on a higher probability, which would slightly reduce the information of word-initial [θ].

**Figure 4.**
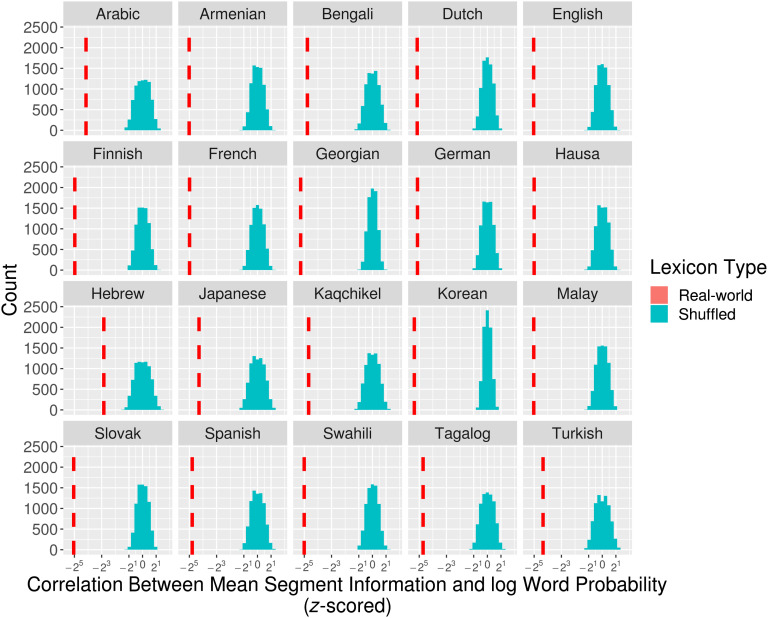
**Distribution for Pearson’s correlation between log word probability and mean token-based segmental information for 10,000 shuffled variants of the real-world lexicons.** The *x*-axis shows the number of standard deviations from the mean correlation in frequency-shuffled variants (in log_2_ scale) and the red dashed lines indicate the correlation in the real-world lexicons. The real-world lexicons show a significantly stronger correlation relative to shuffled variants.

For each language, we compared the Pearson’s correlation between mean segmental information and log word probability in 10,000 probability-shuffled lexicons against the correlation found in the real-word lexicon. In all cases, the correlation in the real-world lexicon was significantly stronger (3+ standard deviations, *p* < .001) than in the shuffled lexicons (Figure 4), indicating that the strength of the real-world correlation is greater than would be expected by chance. This suggests that the real-world lexicons have evolved such that less-probable words have segment sequences that preferentially eliminate higher probability competitors across the word-form.

### Segmental Information Distribution Within Words

The patterns we have presented so far show that less-probable words contain relatively higher information segments. If the lexicon is structured for incremental processing, this bias should be greatest at word beginnings, where high-information segments can better offset lower word probability. If less-probable words evolve higher segment information early in the word, mean segment information should be relatively higher when calculated in the forward than reverse order for less-probable words. Likewise, less-probable words should have earlier relative uniqueness-points in the forward-order than in the reverse. As an example, the less-probable word *thwart* has a high mean segment information and an early uniqueness-point due to the rarity of its initial segments, while in the reverse lexicon, these values are less extreme because of the large set of words that end in [aɹt].

To test this, we compared the mean segment information and relative uniqueness-point for words in each actual lexicon against those in the reverse-order lexicon. Each word in the reverse lexicon is the same as its forward counterpart in length and segment composition, but the set of cohorts defined by each successive segment is different. For example, the initial cohort for *thwart* comprises the relatively few words beginning with [θ], while in the reverse lexicon it comprises the many words ending in [t]. In these studies, we computed mean segment information only up to the uniqueness-point, with the result that the same word can have different mean segment information when calculated in the forward or backward lexicon. Note that because our measure of segment information treats segments as abstract symbols defining a network, using a reverse lexicon is licit even though it creates segment sequences that may not be pronounceable. We fit linear mixed-effects models over a pooled dataset of all languages to predict the forward and reverse values by (a) word probability, (b) a binary factor for lexicon order (reverse-order vs. forward-order), and (c) their interaction, with random intercepts and slopes for word and language nested within family. In all models, the interaction between order and word probability was significant with the expected sign, supporting the prediction that less-probable words have higher early segmental information in the actual, as opposed to the reverse lexicons (Tables 6, 7, 8 in the Supplemental Materials; King & Wedel, 2020).

To confirm these differences at the word-level (as opposed to just across the lexicons as a whole), we constructed linear mixed-effects models to predict the *difference* in forward- and reverse-order measures within each word, by subtracting the reverse from the forward-lexicon value. These models provided the same outcomes, supporting the hypothesis that less-probable words tend to evolve higher segment information early (Tables 9, 10, 11 in the Supplemental Materials; King & Wedel, 2020). Using this approach, we additionally carried out regressions within each individual language (Figures 5, 6, 7 in the Supplemental Materials; King & Wedel, 2020). We found that the majority, but not all languages showed the significant effects apparent within the pooled dataset; see Discussion.

## DISCUSSION

We have presented evidence that segments in less-probable words convey more disambiguating information in incremental processing. Further, in many languages this positive correlation is concentrated at word-beginnings, where the potential difference in segmental information is greatest. These findings contribute compelling evidence that lexicons are optimized efficient communication overall within the constraints of the language processing system (Dautriche et al., 2017; Ferrer i Cancho & Solé, 2003; Gibson et al., 2019; Mahowald et al., 2018; Meylan & Griffiths, 2017; Piantadosi et al., 2011, 2012).

### Evolution of Word-Forms in the Lexicon

How might these lexical patterns arise? Evidence from corpus studies suggest that less informative segments are more likely to be shortened or deleted in speech (Cohen Priva, 2015, 2017), and are more likely to be replaced by similar sounds over time (Wedel, Kaplan, & Jackson, 2013). Parallel evidence shows that more-probable words are more likely to shorten (Bybee & Hopper, 2001; Kanwal et al., 2017) and become more similar to other words (Frauenfelder, Baayen, & Hellwig, 1993; Mahowald et al., 2018). Conversely, segments that provide more information tend to be pronounced with greater clarity (Aylett & Turk, 2004, 2006; Buz, Jaeger, & Tanenhaus, 2014; Nelson & Wedel, 2017; Sano, 2017; Seyfarth, Buz, & Jaeger, 2016; van Son & Pols, 2003; van Son & van Santen, 2005; Wedel, Nelson, & Sharp, 2018), and are more likely to persist in a language over time (Wedel et al., 2013).

Because more-probable words require less segmental information to be accurately understood (reviewed in Hall et al., 2016), these considerations predict that segments in more-probable words should be more rapidly lost, or replaced over time with more-frequent segments (see discussion in Bybee & Hopper, 2001; Piantadosi et al., 2011). Because of this asymmetry, over long time periods more-probable words should drift into denser, phonotactically probable cohorts, while less-probable words should preferentially retain less-common segment sequences, leaving them in sparser regions of the lexicon’s network structure.

All languages in the dataset show a significant correlation between lower word probability and greater incremental segment information. Why do some languages fail to show a skew toward higher segment information at beginnings of less-probable words? Many of those particular languages have constraints that enforce denser lexical networks: for example, word-forms in Hebrew and Arabic are based in tri-consonantal roots that constrain lexicon size (Ussishkin, 2005); words in Kaqchikel are based on single syllable roots (Bennett, 2016); words in Tagalog and Malay have simple phonotactics and tend to be bi-syllabic (Blust, 2007); Swahili, likewise, has simple phonotactics and a preference for bisyllabic word stems (Mohamed, 2001). These language-specific constraints on word-forms result in denser lexical networks, which should inhibit loss of information late in word-forms. Initial work indicates a significant link between denser lexical networks and maintenance of late segment information in less-probable words.

### Broader Implications

Zipf’s law of abbreviation is strikingly consistent across a wide range of tested languages (Bentz & Ferrer i Cancho, 2016). Likewise, we find a similar pattern of correlation between word probability and segment information across a diverse set of languages. The fact that we see similar correlations in each of these languages suggests that like Zipf’s law of abbreviation, this may also be a robust, “statistically-universal” property of human languages (Dryer, 1998). Together with the evidence that word-forms are shaped for efficient production by speakers (Dautriche, 2015; Mahowald et al., 2018; Meylan & Griffiths, 2017), the findings here support a broader trend of linguistic evolution toward systems that benefit both speakers and listeners, in which modulation of segment number and segment composition in words are complementary parts of this larger process.

## ACKNOWLEDGMENTS

The authors would like to thank Roger Levy and the two anonymous reviewers for their insightful comments and suggestions during the preparation of this article. The authors would also like to thank the attendees of CUNY 2016 and EvoLang XII for useful discussion and feedback.

## FUNDING INFORMATION

AK, National Science Foundation (NSF), Award ID: Graduate Research Fellowship (2016233374).

## AUTHOR CONTRIBUTIONS

AK: Conceptualization: Lead; Data curation: Lead; Formal analysis: Equal; Methodology: Equal; Resources: Lead; Visualization: Lead; Writing—Original Draft: Equal; Writing—Review & Editing: Equal. AW: Formal analysis: Equal; Methodology: Equal; Supervision: Equal; Writing—Original Draft: Equal; Writing—Review & Editing: Equal.

## Notes

^1^ Though the use of larger contextual windows has been shown to provide a potentially better fit between word probability and word length (Piantadosi et al., 2011), context-free probability is strongly correlated with probability measured over larger context window sizes (see Cohen Priva & Jaeger, 2018, for more).^2^ We added end-of-word boundary symbols to all words, setting the uniqueness-point to be the word length plus one for words that do not have a word-internal uniqueness-point, e.g., *cat*, given the existence of *catalog*.^3^ There is a significant, negative correlation between word length and mean segment information in each language. Variance inflation factor (vif) scores were below an acceptable threshold (<2) in all languages (see O’brien, 2007, for discussion of vif).^4^ However, here it is not precisely equivalent, because we subtract a word’s frequency from the calculation of information for its own segments.^5^ Here we use the unmodified form of segment information (Equation 2) because the built-in correlation between word probability and token-based segment information is the same for the real-world and shuffled lexicons.
