# Supplementary material for: Greater Early Disambiguating Information for Less-Probable Words: The Lexicon Is Shaped by Incremental Processing
Source: Open Mind (Camb). 2020 Mar 1;4:1–12. doi: 10.1162/opmi_a_00030 (PMC7323847; doi:10.1162/opmi_a_00030)
Supplement: Supplementary file 1 [file opmi-04-1-s001.pdf]

SUPPLEMENTAL MATERIAL

Data Sources

Table 1: Description of language corpora.

Language	Source	Parsing level	Num. Word Types
Arabic	Canavan and Graff (1997)	Lemma	2851
Armenian	Khurshudian and Daniel (2009)	Lemma	10000
Bengali	Kilgarriff et al. (2014)	Lemma	10000
Dutch	Baayen, Piepenbrock, and Gulikers (1995)	Lemma	10000
English	Weide (1996) (forms) and Davies (2008) (freq.)	Lemma	7741
Finnish	*"Finnish Text Collection"* (2005)	Lemma	9390
French	New, Pallier, Ferrand, and Matos (2001)	Lemma	10000
Georgian	Gippert and Tandashvili (2012)	Lemma	10000
German	Baayen et al. (1995)	Lemma	10000
Hausa	Kilgarriff et al. (2014)	None[5]	10000
Hebrew	Linzen (2009)	Lemma	4442
Japanese	Canavan and Graff (1997)	Lemma	1511
Kaqchikel	Tang and Bennett (2018)	None[5]	5943
Korean	Lee (2006)	Lemma	10000
Malay	Lison and Tiedemann (2016)	None[5]	10000
Slovak	Krajčovič (1988)	Lemma	10000
Spanish	Mendonca, Graff, and DiPersio (2009)	Lemma	10000
Swahili	Hurskainen (2004)	Lemma	10000
Tagalog	Goldhahn and Quasthoff (2012)	None[5]	10000
Turkish	Sak, Güngör, and Saraçlar (2008)	Lemma	10000

Linear Mixed-effects Models (Sections 1-3)

[5] Corpora further cleaned using regular expression to exclude forms that do not conform to language's spelling conventions.

Table 2: Linear models to predict log word probability, given mean token-based segment information and word length. All factors have been z-scored.

		Estimate	Std. Error	t value	Pr(>|t|)
Arabic	Mean Seg. Info. (token)	-0.1839	0.0205	-8.95	0.0001
	Word Length	-0.3651	0.0205	-17.77	0.0001
Armenian	Mean Seg. Info. (token)	-0.2752	0.0115	-23.86	0.0001
	Word Length	-0.3816	0.0115	-33.09	0.0001
Bengali	Mean Seg. Info. (token)	-0.2386	0.0120	-19.91	0.0001
	Word Length	-0.3385	0.0120	-28.25	0.0001
Dutch	Mean Seg. Info. (token)	-0.2438	0.0111	-21.90	0.0001
	Word Length	-0.4506	0.0111	-40.47	0.0001
English	Mean Seg. Info. (token)	-0.1440	0.0121	-11.91	0.0001
	Word Length	-0.3424	0.0121	-28.32	0.0001
Finnish	Mean Seg. Info. (token)	-0.2506	0.0115	-21.78	0.0001
	Word Length	-0.3685	0.0115	-32.03	0.0001
French	Mean Seg. Info. (token)	-0.1720	0.0110	-15.63	0.0001
	Word Length	-0.3442	0.0110	-31.28	0.0001
Georgian	Mean Seg. Info. (token)	-0.2797	0.0111	-25.13	0.0001
	Word Length	-0.2674	0.0111	-24.03	0.0001
German	Mean Seg. Info. (token)	-0.2910	0.0111	-26.15	0.0001
	Word Length	-0.4370	0.0111	-39.27	0.0001
Hausa	Mean Seg. Info. (token)	-0.3734	0.0114	-32.78	0.0001
	Word Length	-0.3340	0.0114	-29.33	0.0001
Hebrew	Mean Seg. Info. (token)	-0.1078	0.0182	-5.94	0.0001
	Word Length	-0.2142	0.0182	-11.79	0.0001
Japanese	Mean Seg. Info. (token)	-0.1145	0.0258	-4.44	0.0001
	Word Length	-0.3536	0.0258	-13.72	0.0001
Kaqchikel	Mean Seg. Info. (token)	-0.1952	0.0140	-13.97	0.0001
	Word Length	-0.2809	0.0140	-20.10	0.0001
Korean	Mean Seg. Info. (token)	-0.1448	0.0102	-14.25	0.0001
	Word Length	-0.2049	0.0102	-20.17	0.0001
Malay	Mean Seg. Info. (token)	-0.3146	0.0113	-27.75	0.0001
	Word Length	-0.3338	0.0113	-29.45	0.0001
Slovak	Mean Seg. Info. (token)	-0.2802	0.0110	-25.41	0.0001
	Word Length	-0.3102	0.0110	-28.13	0.0001
Spanish	Mean Seg. Info. (token)	-0.2947	0.0116	-25.43	0.0001
	Word Length	-0.3038	0.0116	-26.21	0.0001
Swahili	Mean Seg. Info. (token)	-0.2097	0.0104	-20.07	0.0001
	Word Length	-0.2015	0.0104	-19.29	0.0001
Tagalog	Mean Seg. Info. (token)	-0.1984	0.0114	-17.37	0.0001
	Word Length	-0.3808	0.0114	-33.34	0.0001
Turkish	Mean Seg. Info. (token)	-0.2305	0.0103	-22.40	0.0001

	Word Length	-0.2327	0.0103	-22.61	0.0001

Table 3: Linear models to predict log word probability, given mean type-based segment information and word length. All factors have been z-scored.

		Estimate	Std. Error	t value	Pr(>|t|)
Arabic	Mean Seg. Info. (type)	-0.1166	0.0216	-5.40	0.0001
	Word Length	-0.3378	0.0216	-15.65	0.0001
Armenian	Mean Seg. Info. (type)	-0.2299	0.0121	-18.95	0.0001
	Word Length	-0.3661	0.0121	-30.18	0.0001
Bengali	Mean Seg. Info. (type)	-0.2325	0.0124	-18.82	0.0001
	Word Length	-0.3417	0.0124	-27.65	0.0001
Dutch	Mean Seg. Info. (type)	-0.2298	0.0114	-20.18	0.0001
	Word Length	-0.4481	0.0114	-39.35	0.0001
English	Mean Seg. Info. (type)	-0.1037	0.0131	-7.91	0.0001
	Word Length	-0.3361	0.0131	-25.63	0.0001
Finnish	Mean Seg. Info. (type)	-0.2545	0.0117	-21.77	0.0001
	Word Length	-0.3760	0.0117	-32.15	0.0001
French	Mean Seg. Info. (type)	-0.1769	0.0115	-15.42	0.0001
	Word Length	-0.3568	0.0115	-31.09	0.0001
Georgian	Mean Seg. Info. (type)	-0.2887	0.0113	-25.47	0.0001
	Word Length	-0.2798	0.0113	-24.68	0.0001
German	Mean Seg. Info. (type)	-0.2548	0.0115	-22.23	0.0001
	Word Length	-0.4235	0.0115	-36.95	0.0001
Hausa	Mean Seg. Info. (type)	-0.3900	0.0121	-32.26	0.0001
	Word Length	-0.3681	0.0121	-30.45	0.0001
Hebrew	Mean Seg. Info. (type)	-0.0805	0.0191	-4.22	0.0001
	Word Length	-0.2085	0.0191	-10.93	0.0001
Japanese	Mean Seg. Info. (type)	-0.1163	0.0263	-4.42	0.0001
	Word Length	-0.3601	0.0263	-13.68	0.0001
Kaqchikel	Mean Seg. Info. (type)	-0.1872	0.0147	-12.72	0.0001
	Word Length	-0.2921	0.0147	-19.84	0.0001
Korean	Mean Seg. Info. (type)	-0.1276	0.0102	-12.51	0.0001
	Word Length	-0.2005	0.0102	-19.67	0.0001
Malay	Mean Seg. Info. (type)	-0.3629	0.0117	-30.89	0.0001
	Word Length	-0.3791	0.0117	-32.27	0.0001
Slovak	Mean Seg. Info. (type)	-0.2749	0.0115	-23.94	0.0001
	Word Length	-0.3217	0.0115	-28.01	0.0001
Spanish	Mean Seg. Info. (type)	-0.2589	0.0124	-20.91	0.0001
	Word Length	-0.2997	0.0124	-24.21	0.0001
Swahili	Mean Seg. Info. (type)	-0.2028	0.0109	-18.63	0.0001
	Word Length	-0.2151	0.0109	-19.76	0.0001
Tagalog	Mean Seg. Info. (type)	-0.1559	0.0121	-12.86	0.0001
	Word Length	-0.3662	0.0121	-30.22	0.0001
Turkish	Mean Seg. Info. (type)	-0.2106	0.0106	-19.81	0.0001

	Word Length	-0.2386	0.0106	-22.44	0.0001

Table 4: Linear models to predict relative uniqueness point position, given log word probability. Factor for log Word Probability has been z-scored.

		Estimate	Std. Error	t value	Pr(>|t|)
Arabic	log Word Prob.	0.2964	0.0179	16.56	0.0001
Armenian	log Word Prob.	0.3149	0.0095	33.18	0.0001
Bengali	log Word Prob.	0.2927	0.0096	30.60	0.0001
Dutch	log Word Prob.	0.4018	0.0092	43.86	0.0001
English	log Word Prob.	0.3340	0.0107	31.17	0.0001
Finnish	log Word Prob.	0.3627	0.0096	37.70	0.0001
French	log Word Prob.	0.3275	0.0094	34.66	0.0001
Georgian	log Word Prob.	0.3147	0.0095	33.16	0.0001
German	log Word Prob.	0.3807	0.0092	41.17	0.0001
Hausa	log Word Prob.	0.3454	0.0094	36.80	0.0001
Hebrew	log Word Prob.	0.1389	0.0165	8.41	0.0001
Japanese	log Word Prob.	0.3868	0.0237	16.30	0.0001
Kaqchikel	log Word Prob.	0.2849	0.0124	22.90	0.0001
Korean	log Word Prob.	0.2180	0.0098	22.33	0.0001
Malay	log Word Prob.	0.3701	0.0093	39.84	0.0001
Slovak	log Word Prob.	0.2901	0.0096	30.30	0.0001
Spanish	log Word Prob.	0.2655	0.0096	27.54	0.0001
Swahili	log Word Prob.	0.2245	0.0097	23.04	0.0001
Tagalog	log Word Prob.	0.3086	0.0095	32.44	0.0001
Turkish	log Word Prob.	0.2587	0.0097	26.77	0.0001

Table 5: Linear models to predict non-relative uniqueness point position, given log word probability and word length. Independent factors have been z-scored.

		Estimate	Std. Error	t value	Pr(>|t|)
Arabic	log Word Probability	0.1079	0.0152	7.10	0.0001
	Word Length	0.6478	0.0152	42.64	0.0001
Armenian	log Word Probability	0.1319	0.0070	18.75	0.0001
	Word Length	0.7468	0.0070	106.15	0.0001
Bengali	log Word Probability	0.1293	0.0072	18.02	0.0001
	Word Length	0.7254	0.0072	101.16	0.0001
Dutch	log Word Probability	0.1856	0.0079	23.50	0.0001
	Word Length	0.6968	0.0079	88.23	0.0001
English	log Word Probability	0.1123	0.0094	11.92	0.0001
	Word Length	0.6266	0.0094	66.50	0.0001
Finnish	log Word Probability	0.1985	0.0076	26.25	0.0001
	Word Length	0.7219	0.0076	95.46	0.0001
French	log Word Probability	0.1409	0.0079	17.84	0.0001
	Word Length	0.6685	0.0079	84.64	0.0001
Georgian	log Word Probability	0.2286	0.0080	28.49	0.0001
	Word Length	0.5904	0.0080	73.57	0.0001
German	log Word Probability	0.1866	0.0077	24.09	0.0001
	Word Length	0.6952	0.0077	89.72	0.0001
Hausa	log Word Probability	0.2117	0.0068	30.97	0.0001
	Word Length	0.7309	0.0068	106.91	0.0001
Hebrew	log Word Probability	0.0580	0.0141	4.10	0.0001
	Word Length	0.5561	0.0141	39.34	0.0001
Japanese	log Word Probability	0.1352	0.0239	5.67	0.0001
	Word Length	0.5013	0.0239	21.01	0.0001
Kaqchikel	log Word Probability	0.1471	0.0106	13.85	0.0001
	Word Length	0.6078	0.0106	57.25	0.0001
Korean	log Word Probability	0.1221	0.0094	12.97	0.0001
	Word Length	0.3721	0.0094	39.52	0.0001
Malay	log Word Probability	0.2345	0.0074	31.62	0.0001
	Word Length	0.6794	0.0074	91.61	0.0001
Slovak	log Word Probability	0.1913	0.0081	23.50	0.0001
	Word Length	0.5991	0.0081	73.58	0.0001
Spanish	log Word Probability	0.1620	0.0072	22.35	0.0001
	Word Length	0.7002	0.0072	96.61	0.0001
Swahili	log Word Probability	0.1527	0.0086	17.74	0.0001
	Word Length	0.5176	0.0086	60.15	0.0001
Tagalog	log Word Probability	0.1299	0.0077	16.89	0.0001
	Word Length	0.6953	0.0077	90.38	0.0001
Turkish	log Word Probability	0.1712	0.0089	19.26	0.0001

	Word Length	0.4735	0.0089	53.28	0.0001

Comparisons between Forward and Reversed-order lexicons (Section 5)

Interaction of Word Probability with Lexicon-order in Linear Mixed-effects Models As described in the main text, we used linear mixed effects models to test the interaction of word probability with lexicon-order on mean token-based segment information, mean type-based segment information, and relative uniqueness-point. In these models, less probable words in the original forward-order lexicons were shown to have significantly greater mean segment information (token and type), as well as earlier uniqueness-points, when compared to their reverse-ordered versions. This suggests that when we abstract away from individual language differences, less probable words tend to evolve relatively higher segment information early rather than late in their forms.

Table 6. Linear mixed-effect model for predicting mean token-based segment information. The model showed a significant, negative interaction between word probability and the forward-order lexicon, indicating a stronger effect of word probability in the original lexicon compared to the reversed-ordered version. p values determined via the Satterthwaite's degrees of freedom method implemented in the LMERTEST library (Kuznetsova et al., 2017). Models were fit with correlation between random factors removed to ensure model convergence (for more description, see Bates and Walker 2015).

A. Fixed Effects:

	Estimate	Std. Error	t value	Pr(>|t|)
(Intercept)	0.028	0.001	0.010	0.992
log Word Prob.	-0.211	0.018	-11.512	0.001
Forward-order	0.086	0.185	0.465	0.649
Word Prob.:Forward	-0.043	0.017	-2.550	0.035

B. Random Effects:

	Name	Variance	Std.Dev.
Language:Word	(Intercept)	0.269	0.512
Family	(Intercept)	0.001	0.003
	log Prob.	0.001	0.001
	Forward	0.001	0.010
	Reverse	0.111	0.334
	log Prob.:Forward	0.001	0.023
	log Prob.:Reverse	0.001	0.001
Language:Family	(Intercept)	0.089	0.297
	log Prob.	0.001	0.012
	Forward	0.015	0.281
	Reverse	0.079	0.123
	log Prob.:Forward	0.001	0.227
	log Prob.:Reverse	0.001	0.173

By-word Difference in Linear Mixed-effects Models To assess whether these trends are significant within each word as opposed to the lexicons treated as a whole, we subtracted the reverse-order value for each measure from the forward-order value for each word, and

Table 7. Linear mixed-effect model for predicting mean type-based segment information. The model showed a significant, negative interaction between word probability and the forward-order lexicon, indicating that early segments in less probable words exclude more competing word types in the original, forward-ordered lexicon compared to the reversed-order version.

A. Fixed Effects:

	Estimate	Std. Error	t value	Pr(>|t|)
(Intercept)	-0.052	0.193	-0.271	0.790
log Word Prob.	-0.031	0.015	-2.082	0.071
Forward-order	0.114	0.222	0.516	0.614
Word Prob.:Forward	-0.050	0.020	-2.499	0.036

B. Random Effects:

	Name	Variance	Std.Dev.
Language:Word	(Intercept)	0.306	0.553
Family	(Intercept)	0.001	0.008
	log Prob.	0.001	0.001
	Forward	0.001	0.017
	Reverse	0.159	0.399
	log Prob.:Forward	0.001	0.008
	log Prob.:Reverse	0.001	0.019
Language:Family	(Intercept)	0.088	0.297
	log Prob.	0.001	0.011
	Forward	0.132	0.363
	Reverse	0.039	0.197
	log Prob.:Forward	0.001	0.012
	log Prob.:Reverse	0.001	0.025

tested for a significant effect of word probability on this difference measure. For the tests reported in this section, we excluded short words (¡5 segments) because the ends of short words are necessarily close to their beginnings, and therefore we do not expect as large a difference for forward and reverse measures. Language was nested within language family as random intercepts, with log word probability as a random slope on both. In these models, lower word probability was significantly predictive of greater mean segment information (token and type), as well as earlier relative uniqueness-point difference within each word, when measured as the difference in the forward versus reverse-order measures (Tables 9, 10, 11) .

Figs. 5, 6, 7 illustrate the predictive value of log word probability on the differences between the forward and reversed-ordered lexicons (see 8 for summary). For most of the languages in the dataset, as predicted, there was a significant, negative effect of log word probability on the difference between forward and reversed-order for token and type mean segment information and a significant, positive effect on difference in relative uniqueness-point.

BY-WORD DIFFERENCE IN LINEAR MIXED-EFFECTS MODELS INCLUDING ALL WORD LENGTHS

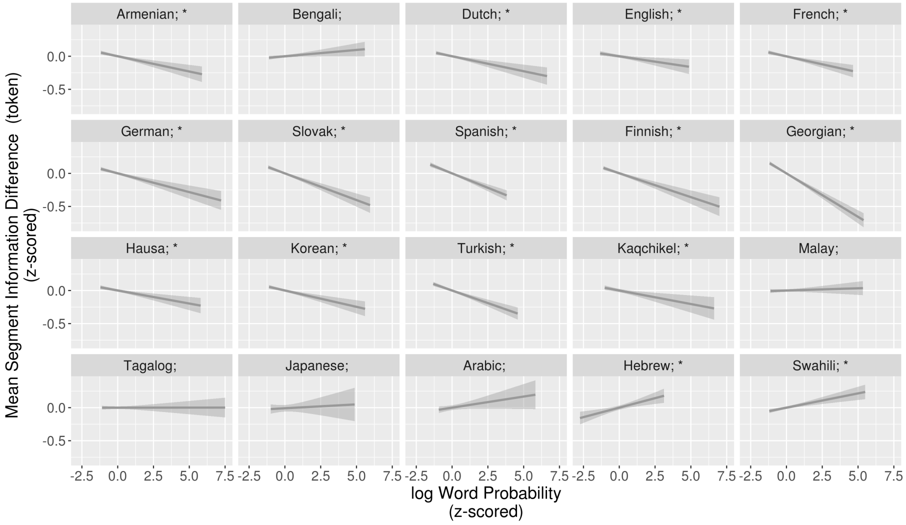

Figure 5. Relationship between log word probability and forward-reverse difference for mean token-based segment information; significant effects ($p < .05$) marked with asterisks after language name. For most languages, less probable words have a significantly greater mean token-based information. Note, Hebrew and Swahili have a significant effect in the opposite direction (see Discussion).

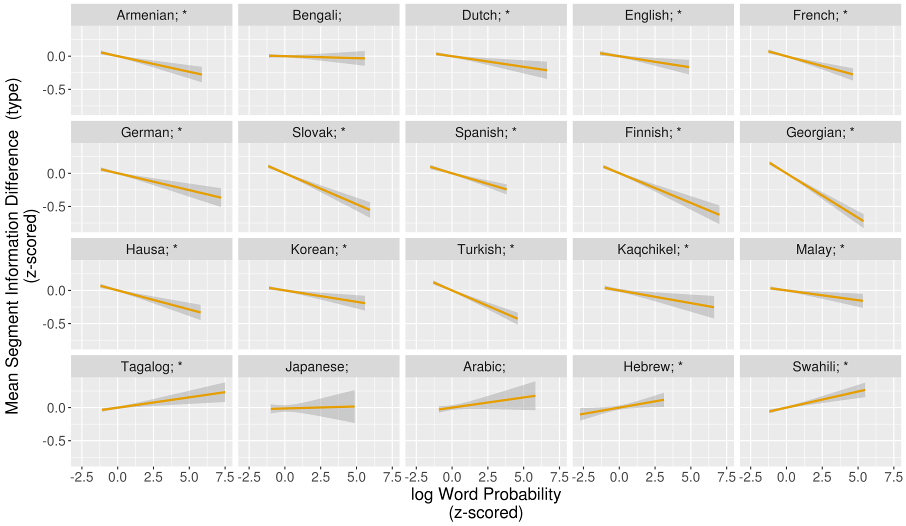

Figure 6. Relationship between log word probability and forward-reverse difference for mean type-based segment information; significant effects marked with asterisks after language name. For most languages, less probable words have a significantly greater mean type-based information. Note, Tagalog, Hebrew and Swahili have a significant effect in the opposite direction (see Discussion).

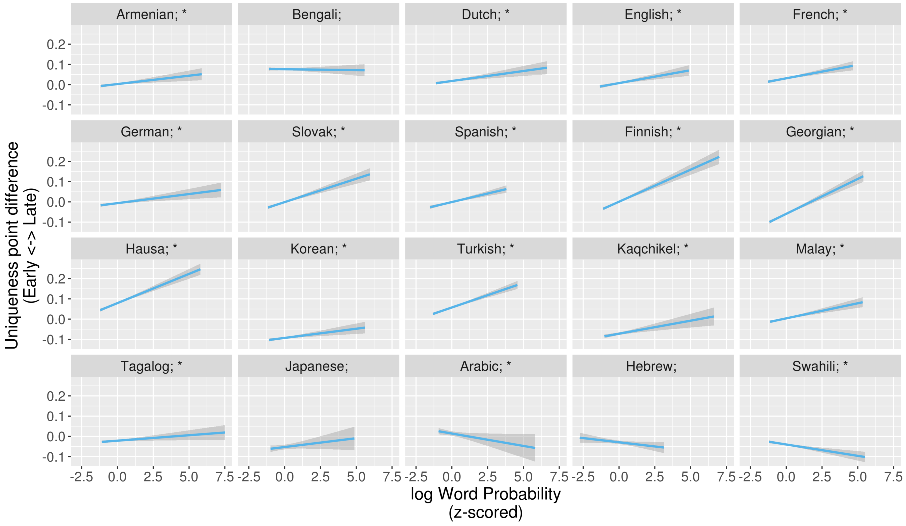

Figure 7. Relationship between log word probability and forward-reverse difference for relative uniqueness-point; significant effects marked with asterisks after language name. For most languages, less probable words possess earlier uniqueness-points when compared to their reversed counterpart. Note, Arabic and Swahili have a significant effect in the opposite direction (see Discussion).

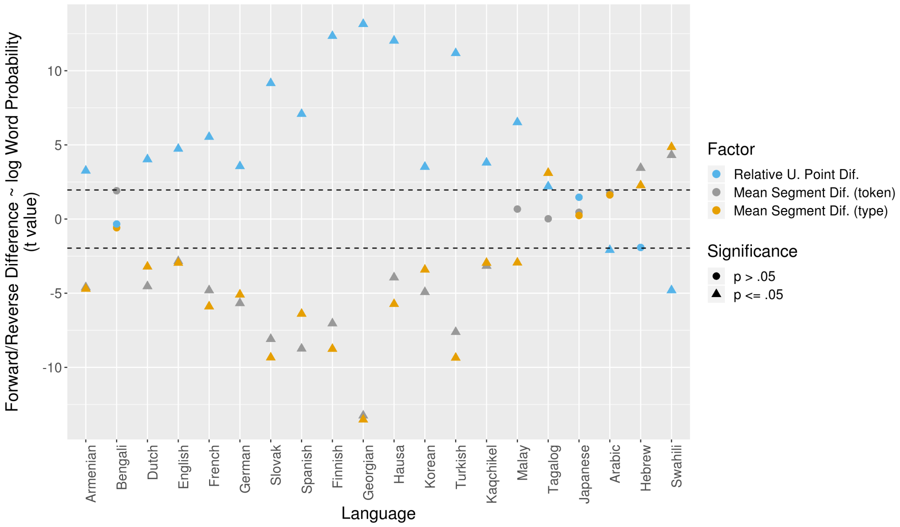

Figure 8. Summary of models for by-word difference measures between forward and reversed-order word-forms. The y-axis represents the t value for log Word Probability in linear models to predict the indicated factor. The dashed lines represent $+/-$ 1.96, beyond which $p < .05$. If less probable words have higher early segment information in the forward than the reverse-order lexicons, the subtraction of the reverse value from the forward value for each word should produce a negative correlation, i.e., a negative t value, for word probability with mean segment information (token and type), and a positive correlation with relative uniqueness-point. This is the case for most languages in the dataset (see Discussion).

Table 8. Linear mixed-effect model for predicting relative uniqueness point. The model showed a significant, positive interaction between word probability and the forward-order lexicon, indicating that less probable words have earlier uniqueness points in the original, forward-ordered lexicon when compared to their reverse-ordered version.

A. Fixed Effects:

	Estimate	Std. Error	t value	Pr(>|t|)
(Intercept)	-0.027	0.210	-0.129	0.897
log Word Prob.	0.260	0.019	13.498	0.001
Forward-order	-0.030	0.168	-0.178	0.859
Word Prob.:Forward	0.064	0.014	4.346	0.001

B. Random Effects:

	Name	Variance	Std.Dev.
Language:Word Family	(Intercept)	0.228	0.001
	(Intercept)	0.001	0.003
	log Prob.	0.001	0.001
	Forward	0.001	0.003
	Reverse	0.001	0.003
	log Prob.:Forward	0.001	0.001
	log Prob.:Reverse	0.001	0.001
Language:Family	(Intercept)	0.001	0.018
	log Prob.	0.001	0.038
	Forward	0.653	0.808
	Reverse	0.427	0.653
	log Prob.:Forward	0.001	0.021
	log Prob.:Reverse	0.001	0.034

To ask if these results were dependent on the exclusion of the shortest words (¡5 segments), we fit additional models including word length and its interaction with word probability as fixed and random effects. In these models, word probability remained significantly predictive of the dependent measures, and in addition, the interactions between word length and word probability were significant in the same direction as the effect of word probability, indicating that as expected, shorter words show less of a difference between the forward and reverse measures (see Tables 12, 13, 14).

Table 9. Linear mixed-effect model for predicting Forward-Reverse difference for mean token-based segment information for words of length 5 or greater. The model showed a negative effect of word probability, suggesting that less probable words in the original lexicon show a stronger correlation with mean token-based segment information compared to the reversed-order lexicon.

A. Fixed Effects:

	Estimate	Std. Error	t value	Pr(>|t|)
(Intercept)	0.118	0.068	1.720	0.121
log Word Prob.	-0.049	0.020	-2.452	0.048

B. Random Effects:

	Name	Variance	Std.Dev.
Language:Family	(Intercept)	0.014	0.118
	log Word Prob.	0.002	0.053
Family	(Intercept)	0.023	0.153
	log Word Prob.	0.001	0.022

Table 10. Linear mixed-effect model for predicting Forward-Reverse difference for mean type-based segment information for words of length 5 or greater. The model showed a negative effect of word probability, suggesting that less probable words in the original lexicon show a stronger correlation with mean type-based segment information compared to the reversed-order lexicon.

A. Fixed Effects:

	Estimate	Std. Error	t value	Pr(>|t|)
(Intercept)	0.104	0.057	1.819	0.105
log Word Prob.	-0.056	0.014	-3.907	0.005

B. Random Effects:

	Name	Variance	Std.Dev.
Language:Family	(Intercept)	0.011	0.108
	log Word Prob.	0.002	0.050
Family	(Intercept)	0.016	0.129
	log Word Prob.	0.001	0.018

Table 11. Linear mixed-effect model for predicting Forward-Reverse difference for relative uniqueness point for words of length 5 or greater. There is a positive effect of log word probability, suggesting that less probable word forms in the original lexicon have earlier uniqueness points compared to reversed-order.

A. Fixed Effects:

	Estimate	Std. Error	t value	Pr(>|t|)
(Intercept)	-0.019	0.015	-1.308	0.225
log Word Prob.	0.015	0.003	4.400	0.001

B. Random Effects:

	Name	Variance	Std.Dev.
Language:Family	(Intercept)	0.001	0.034
	log Word Prob.	0.001	0.014
Family	(Intercept)	0.001	0.028
	log Word Prob.	4.343	0.002

Table 12. Linear mixed-effect model for predicting Forward-Reverse difference for mean token-based segment information.

.

A. Fixed Effects:

	Estimate	Std. Error	t value	Pr(>|t|)
(Intercept)	0.046	0.042	1.081	0.313
log Word Prob.	-0.027	0.012	-2.209	0.049
Word Length	0.035	0.035	1.003	0.342
log Word Prob.:Word Length	-0.015	0.006	-2.339	0.032

B. Random Effects:

	Name	Variance	Std.Dev.
Language:Family	(Intercept)	0.011	0.108
	log Word Prob.	0.002	0.049
	Word Length	0.002	0.045
	log Word Prob:Word Length	0.001	0.022
Family	(Intercept)	0.008	0.092
	log Word Prob.	0.001	0.012
	Word Length	0.009	0.097
	log Word Prob:Word Length	7.869	0.002

Table 13. Linear mixed-effect model for predicting Forward-Reverse difference for mean type-based segment information.

A. Fixed Effects:

	Estimate	Std. Error	t value	Pr(>|t|)
(Intercept)	0.059	0.053	1.117	0.300
log Word Prob.	-0.032	0.012	-2.706	0.037
Word Length	0.053	0.039	1.359	0.209
log Word Prob.:Word Length	-0.018	0.007	-2.577	0.039

B. Random Effects:

	Name	Variance	Std.Dev.
Language:Family	(Intercept)	0.011	0.108
	log Word Prob.	0.002	0.045
	Word Length	0.002	0.052
	log Word Prob:Word Length	0.001	0.019
Family	(Intercept)	0.013	0.115
	log Word Prob.	9.419	0.009
	Word Length	0.012	0.112
	log Word Prob:Word Length	0.001	0.010

Table 14. Linear mixed-effect model for predicting Forward-Reverse difference for relative uniqueness point position.

A. Fixed Effects:

	Estimate	Std. Error	t value	Pr(>|t|)
(Intercept)	-0.070	0.054	-1.303	0.226
log Word Prob.	0.046	0.013	3.661	0.002
Word Length	-0.052	0.046	-1.131	0.289
log Word Prob.:Word Length	0.020	0.003	7.848	0.001

B. Random Effects:

	Name	Variance	Std.Dev.
Language:Family	(Intercept)	0.015	0.122
	log Word Prob.	0.002	0.054
	Word Length	0.004	0.069
Family	(Intercept)	0.010	0.103
	log Word Prob.	7.785	0.002
	Word Length	0.015	0.125
